# CR reprograms acetyl‐CoA metabolism and induces long‐chain acyl‐CoA dehydrogenase and CrAT expression

**DOI:** 10.1111/acel.13266

**Published:** 2020-10-26

**Authors:** Volha Mezhnina, Ryan Pearce, Allan Poe, Nikkhil Velingkaar, Artem Astafev, Oghogho P. Ebeigbe, Kuldeep Makwana, Yana Sandlers, Roman V. Kondratov

**Affiliations:** ^1^ Center for Gene Regulation in Health and Disease and Department of Biological Geological and Environmental Sciences Cleveland State University Cleveland Ohio USA; ^2^ Department of Chemistry Cleveland State University Cleveland Ohio USA

**Keywords:** aging, circadian rhythms, diet, fat, gene expression, metabolism

## Abstract

Calorie restriction (CR), an age delaying diet, affects fat oxidation through poorly understood mechanisms. We investigated the effect of CR on fat metabolism gene expression and intermediate metabolites of fatty acid oxidation in the liver. We found that CR changed the liver acylcarnitine profile: acetylcarnitine, short‐chain acylcarnitines, and long‐chain 3‐hydroxy‐acylcarnitines increased, and several long‐chain acylcarnitines decreased. Acetyl‐CoA and short‐chain acyl‐CoAs were also increased in CR. CR did not affect the expression of CPT1 and upregulated the expression of long‐chain and very‐long‐chain Acyl‐CoA dehydrogenases (LCAD and VLCAD, respectively). The expression of downstream enzymes such as mitochondrial trifunctional protein and enzymes in medium‐ and short‐chain acyl‐CoAs oxidation was not affected in CR. CR shifted the balance of fatty acid oxidation enzymes and fatty acid metabolites in the liver. Acetyl‐CoA generated through beta‐oxidation can be used for ketogenesis or energy production. In agreement, blood ketone bodies increased under CR in a time of the day‐dependent manner. Carnitine acetyltransferase (CrAT) is a bidirectional enzyme that interconverts short‐chain acyl‐CoAs and their corresponding acylcarnitines. CrAT expression was induced in CR liver supporting the increased acetylcarnitine and short‐chain acylcarnitine production. Acetylcarnitine can freely travel between cellular sub‐compartments. Supporting this CR increased protein acetylation in the mitochondria, cytoplasm, and nucleus. We hypothesize that changes in acyl‐CoA and acylcarnitine levels help to control energy metabolism and contribute to metabolic flexibility under CR.

## Graphical abstract

CR upregulated the expression of upstream enzymes, such as VLCAD and LCAD in mitochondria fat oxidation and did not affect the downstream enzymes. These changes were associated with increased short‐chain acyl‐CoAs, including acetyl‐CoA, short‐chain acylcarnitines and CrAT, the enzyme that controls their interconversions. Together, the results suggest that significant reprogramming of liver fat metabolism contributes to metabolic flexibility and signaling in CR liver.

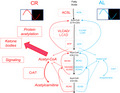

## 1 INTRODUCTION

Increased incidence of metabolic diseases is a significant challenge for modern society. Metabolic syndrome contributes to the development of diabetes, cardiovascular diseases, and cancer (Bonomini et al., 2015; Muoio, 2014). Disruption in lipid homeostasis is one of the hallmarks of metabolic and age related diseases (Johnson & Stolzing, 2019; Savage et al., 2007). Diet is recognized as an important factor affecting disease development and progression. Caloric restriction (CR), a reduction in food intake without malnutrition, is one of the dietary interventions known to have a universally positive effect on metabolism in animal models and humans (Longo et al., 2015). Multiple cellular and physiological systems are affected by CR, and some of these changes may contribute to CR‐induced metabolic health benefits. Shift in energy metabolism is a recognized metabolic adaptation to limited nutrient resources available under CR (Anderson & Weindruch, 2010). Judged by respiratory exchange ratio, AL fed rodents predominantly oxidize carbohydrates around the clock, in contrast with that, CR animals change substrate preference through the day: First few hours after the meal, they oxidize carbohydrates; then, they gradually switch to fatty acid oxidation until the next meal (Bruss et al., 2010). The shift in energy metabolism may be linked with changes in the feeding pattern of CR animals. AL fed animals eat around the clock. CR animals, which are usually fed once per day at the same time, consume the entire meal in roughly 2 h and fast for the next 21–23 h. (Acosta‐Rodríguez et al., 2017; Bruss et al., 2010; Patel et al., 2016; Velingkaar et al., 2020). Changes in fat metabolism under CR have been reported in Drosophila suggesting a conservative response (Katewa et al., 2016).

Fatty acids are oxidized through a chain of reactions that require coordinated activity of several enzymes, which expression and activity in the liver and other tissues are affected by dietary interventions (Barger et al., 2015; Eckel‐Mahan et al., 2013; Kuhla et al., 2014). Little is known if the expression of fatty acid oxidation enzymes is affected in CR. Protein expression has not been reported in CR, and there are several transcriptomic studies with mixed outcomes (reviewed in (Swindell, 2009). Discrepancy of results from different reports may be due to different times of feeding for the experiment and analysis of RNA. The feeding/oxidation cycle has 24‐h periodicity, which implies a possible interaction with circadian rhythms, and this is supported by evidence of a crosstalk between aging, CR, and the circadian clock (Makwana et al., 2019; Patel et al., 2016; Sato et al., 2017). Therefore, the circadian rhythms must be taken into consideration to understand CR‐induced reprogramming of fat metabolism.

It is hypothesized that fat oxidation is increased in CR (Anderson & Weindruch, 2010), and the shift in preference for substrate oxidation is in line with this hypothesis (Bruss et al., 2010). However, molecular events associated with fat oxidation in CR tissues are mostly unknown. Acylcarnitines are intermediate metabolites in beta‐oxidation, and analysis of tissue and blood levels of acylcarnitines is a widely accepted method for evaluating fatty acid oxidation (El‐Gharbawy & Vockley, 2018; Rinaldo et al., 2008). Acylcarnitine profiling has not been performed in mice on CR across the day. Here, we compared acyl‐CoAs, acylcarnitines, and the expression of fatty acid metabolism enzymes across the day in the liver of mice on AL and CR diets. We found that some acylcarnitines oscillated during the day suggesting their production may be regulated by diet and the clock. CR increased expression of several fat catabolism enzymes such as carnitine palmitoyl transferase 2 (CPT2), VLCAD, and LCAD, while CPTI and many downstream enzymes such as mitochondrial trifunctional protein (MTP), medium‐chain acyl‐CoA dehydrogenase (MCAD), short‐chain acyl‐CoA dehydrogenase (SCAD), and acetyl‐CoA acyl transferase 2 (ACAA2) were not affected. Surprisingly, levels of short‐chain acyl‐CoAs (acetyl‐CoA, C4‐CoA and C6‐CoA) and corresponding acylcarnitines were significantly increased in the CR liver. Accumulation of energy rich short‐chain acyl‐CoAs, especially acetyl‐CoA, under limited resources of CR diet was counterintuitive and suggests that they may play additional roles in the mechanisms of CR. We hypothesize that changes in fatty acid oxidation and production of acetyl‐CoA/acetylcarnitine pools coordinate metabolic processes in the liver and may contribute to health benefits of CR.

## 2 RESULTS

### 2.1 CR affected liver acylcarnitine profiles

**Figure 1:**
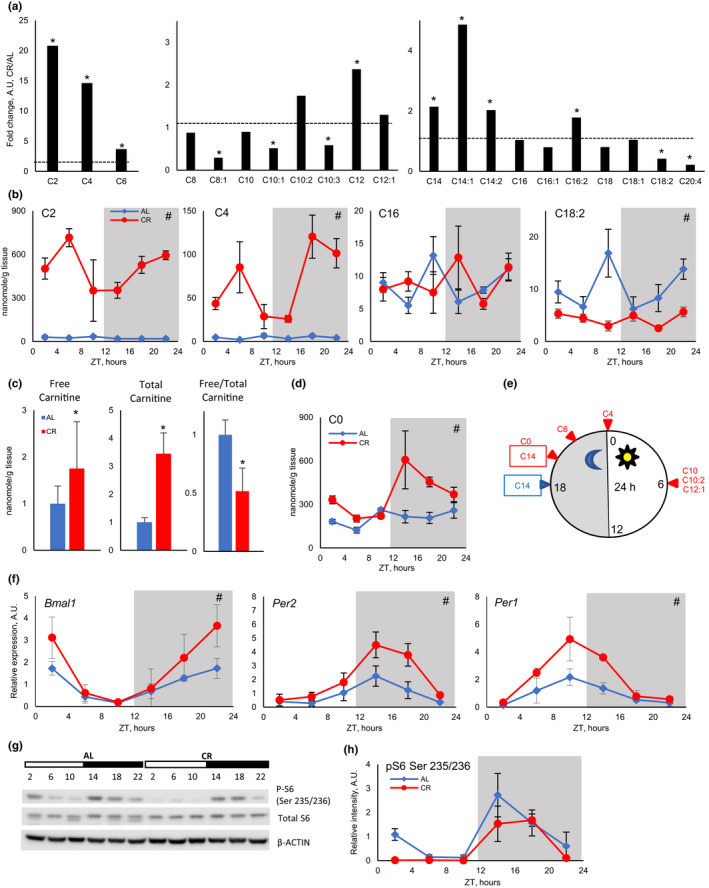
CR increased short‐chain acylcarnitines in the liver. (a) Fold changes in daily average level of short‐chain (left panel), medium‐chain (middle panel), and long‐chain (right panel) acylcarnitines in CR liver (*n* = 24 per diet). Daily average of acylcarnitines in AL liver were set as one, shown as dashed line. Acylcarnitine species are annotated as following [number of carbon atoms]:[number of double bonds]. (b) Daily rhythms of several acylcarnitines in the liver of AL (blue diamonds) and CR (red circles) mice (data represented as average±STD, *n* = 4 per time point per diet). (c) Relative changes in free and total carnitine and ratio of free to total carnitine in AL (blue) and CR (red). (d) Daily rhythms of free carnitine (data represented as average±STD, *n* = 4 per time point per diet). (e) Phase distribution of rhythmic acylcarnitines, AL (blue) and CR (red). (f) Daily rhythms in the expression of circadian clock genes *Bmal1*, *Per2*, and *Per1* (data represented as average±STD, *n* = 3 per time point per diet). (g) Daily rhythms in ribosomal protein S6 phosphorylation and (h) representative Western blot and quantification (data represented as average±STD, *n* = 3 per time point per diet). *—statistically significant difference, *p* < 0.05, by *t* test. #—statistically significant effect of diet, *p* < 0.05, two‐way ANOVA. Shadowed area on the graphs or dark bar above the Western blot represents dark phase of the day, the light was on at ZT0, and the light was off at ZT12. Table S1 contains statistics from ANOVA and student's *t* test for acylcarnitines. Tables S2 and S3 contain statistics for 24‐h and 12‐h rhythm analysis for acylcarnitines, respectively

To study the effect of diet on fatty acid oxidation, CR was implemented to 3‐month old C57B6/J mice. CR mice received 70% of AL food intake to achieve 30% CR for 2 months. CR mice received food at ZT14, 2 h after the light is turned off, which corresponds to the regular feeding time for AL mice. As expected, CR resulted in significantly reduced body weight: Average body weight was 30.6 ± 2.6 g for AL mice and 25.4 ± 1.9 g for CR mice. Blood glucose was also significantly reduced: 173.4 ± 11.5 mg/dl for AL mice and 116.9 ± 13.1 mg/dl for CR mice. After two months of CR, tissues were harvested for the analysis of gene expression and intermediate metabolites in fatty acid oxidation such as acylcarnitines and acyl‐CoA. Fat metabolism is known to be affected by time of feeding and by the circadian clock (Adamovich et al., 2015; Loizides‐Mangold et al., 2017). Therefore, acylcarnitines were analyzed at 6 time points around the clock. We assayed acylcarnitines in the liver of AL and CR mice using LC‐MS/MS. CR has two main effects on acylcarnitine profiles. First, CR significantly affected the level of acylcarnitines, daily averages for short‐, medium‐, and long‐chain acylcarnitines are shown in Figure 1a (relative change) and in Figure S1 (absolute values). Short‐chain acylcarnitines: acetylcarnitine, butyrylcarnitine, and hexanoylcarnitine were significantly upregulated by CR. Daily average levels of acetylcarnitine and butyrylcarnitine were induced by more than tenfolds, and hexanoylcarnitine was induced by about 3‐folds. Medium‐chain (C8‐C12) acylcarnitines were not significantly affected with an exception for 2‐fold induction for C12‐acylcarnitine. The effect of CR on long‐chain acylcarnitine (C14–C20) was not uniform. C14 acylcarnitines were induced by about 2 times, and C16:2 was slightly induced. C16, C16:1, C18, and C18:1 acylcarnitines were not significantly affected. Finally, C18:2‐acylcarnitines and C20:4 acylcarnitines were reduced by CR. CR also resulted in 70% increase in free carnitine and 3‐fold increase in total liver carnitines (Figure 1c). Thus, CR liver either increased the synthesis or the uptake of free carnitine. Interestingly, only 28% of cellular carnitine was free in CR contrary to 50% of free carnitine in AL liver (Figure 1c).

The second major effect of CR was on acylcarnitine daily rhythms. Daily profiles of absolute values for several acylcarnitines are shown in Figure 1b and Figure S2A. While many acylcarnitines demonstrated significant changes throughout the day, only a few of them demonstrated circadian rhythms (Table S2). Figure 1e shows around the clock phase distribution for rhythmic acylcarnitines. AL mice eat around the clock, and they have two major meals, one meal is around ZT12–ZT16 and another meal around ZT20–ZT2 (Velingkaar et al., 2020). We analyzed if some acylcarnitines oscillated with a 12‐h period. Indeed, 9 acylcarnitines demonstrated 12‐h rhythms in AL liver with peaks around the beginning (ZT0) and end (ZT12) of the light period (Table S3 and Figure S2B). Under CR, only three long‐chain acylcarnitines were rhythmic with a 12‐h period (Table S3 and Figure S2B). Free carnitine followed 12‐h rhythms in AL and 24‐h rhythms in CR liver (Figure 1d,e and Figure S2B). Thus, changes in period of acylcarnitine oscillation agreed with changes in food intake pattern.

Circadian clock is implicated in the control of fatty acid metabolism (Adamovich et al., 2015; Loizides‐Mangold et al., 2017). We assayed rhythms in mRNA for circadian clock genes. As it was expected, the expression of *Bmal1*, *Per1*, and *Per2* genes was rhythmic under both diets and rhythms were enhanced by CR (Figure 1f). Phases of clock gene expression were not significantly affected by CR. We also assayed phosphorylation of ribosomal S6 protein as a surrogate marker of mTORC1 activity. In agreement with previous reports (Tulsian et al., 2018), S6 phosphorylation oscillates during the day in the liver on both diets (Figure 1g,h). The peak of S6 phosphorylation was between ZT14 and ZT18 for both diets. Further investigation is required to determine whether the observed changes in acylcarnitine rhythms are linked with changes in the circadian clock or mTOR signaling rhythms.

### 2.2 CR‐induced expression of long‐chain fatty acid beta‐oxidation enzymes in mitochondria

**Figure 2:**
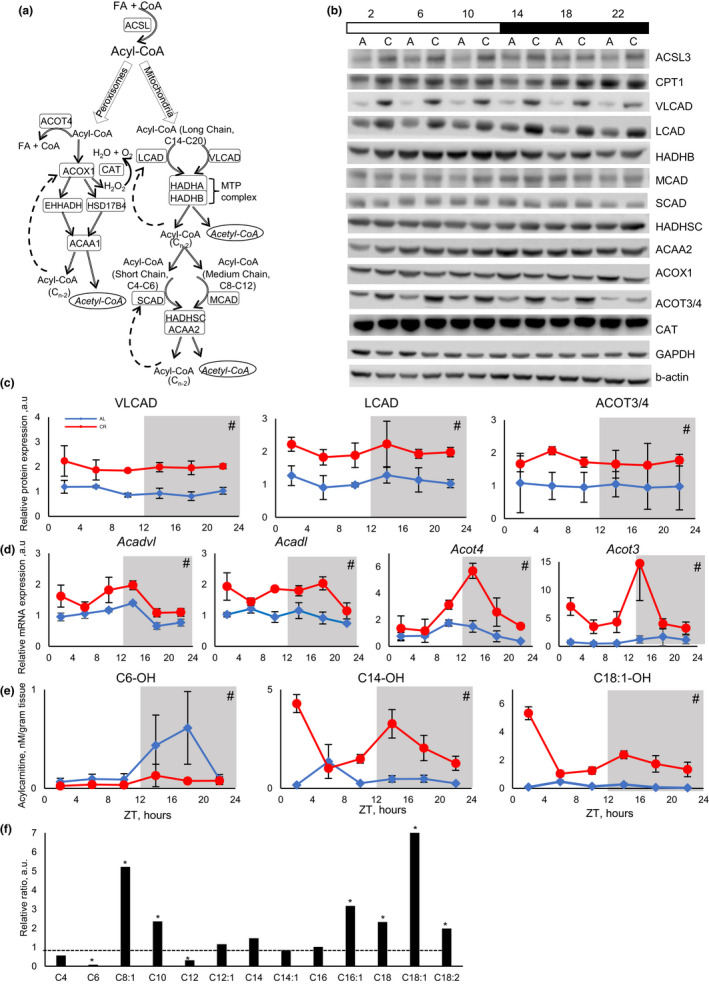
CR resulted in increased oxidation of long‐chain fatty acids in mitochondria. (a) Scheme of fatty acid beta‐oxidation in mitochondria and peroxisomes. Adipose tissue originated long‐chain fatty acids (C14‐C20) are activated to long‐chain Acyl‐CoAs and oxidized through cyclic reaction cascade in mitochondria or peroxisomes. Enzymes controlling corresponding steps in beta‐oxidation are shown in the same color. The first step of mitochondrial beta‐oxidation is catalyzed by four enzymes from the Acyl‐CoA Dehydrogenase (ACAD) family. These enzymes have substrate preferences: very‐long‐chain ACAD C14‐20, long‐chain ACAD (C10–C16), medium‐chain ACAD (C6–C10), and short‐chain ACAD (C4–C6). Long‐chain acyl‐CoAs are oxidized first through the reactions controlled by VLCAD and LCAD, resulting in chain shortening for subsequent oxidation by MCAD and, finally, by SCAD. See further details in the text. (b) Representative Western blotting of beta‐oxidation enzymes in the liver of AL (marked as a) and CR (marked as c) mice, across the day. Every line represents pooled samples (*n* = 3). Light and dark bars represent light and dark phases of the day. (c) Protein and (d) mRNA expression, from left to right: first panels—VLCAD protein and corresponding *Acadvl* gene; second panels—LCAD protein and corresponding *Acadl* gene; (c) third panel—ACOT3/4 protein, and (d) third panel *Acot4* and fourth panel *Acot3* genes (data represented as average±STD, *n* = 3 per time point per diet). (e) Examples of daily rhythms of short‐chain (C6) and long (C14 and C18) hydroxy‐acylcarnitines in the liver of AL (blue lines) and CR (red lines) mice (data represented as average±STD, *n* = 4 per time point per diet). (f) Fold change in ratio between hydroxy‐acylcarnitines and corresponding acylcarnitines in CR liver. The ratio between hydroxy‐acylcarnitine and corresponding acylcarnitine was set as one for AL (*n* = 24 per diet). Acylcarnitine species are annotated as following [number of carbon atoms]:[number of double bonds]. *—statistically significant difference, *p* < 0.05 *t* test. #—statistically significant effect of diet, *p* < 0.05, two‐way ANOVA. Shadowed area on the graphs or dark bar above the Western blot represents dark phase of the day, the light was on at ZT0, and the light was off at ZT12. Tables S4, S5, and S6 contain statistics from anova and student's *t* test for qPCR, Western blot, and hydroxy‐acylcarnitine results, respectively

**Figure 3:**
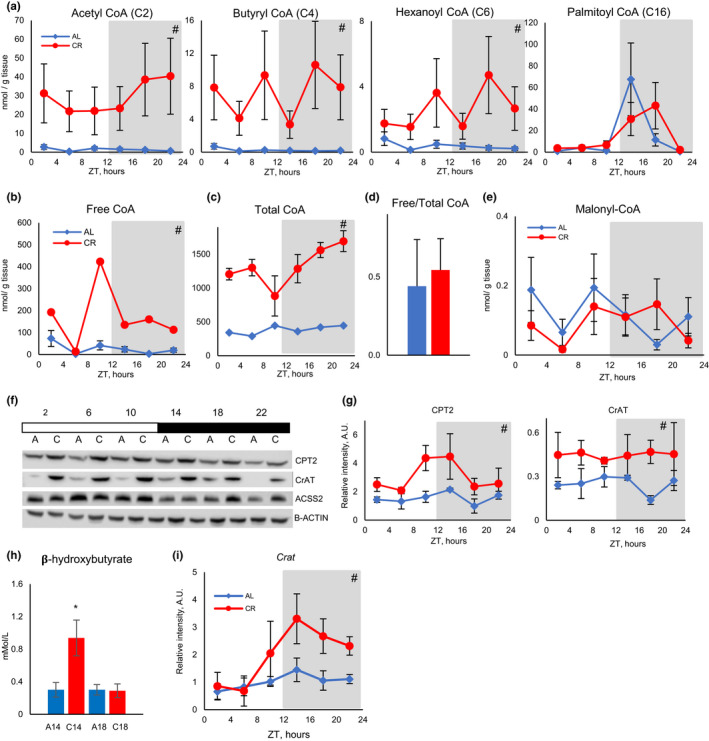
CR increased short‐chain acyl‐CoAs in the liver and blood ketone bodies. (a) Daily rhythms of several acyl‐CoAs in the liver of AL (blue diamonds) and CR (red circles) mice (data represented as average±STD, *n* = 4 per time point per diet). (b) Daily rhythms of free CoA in the liver of AL (blue diamonds) and CR (red circles) mice (data represented as average±STD, *n* = 4 per time point per diet). (c) Daily rhythms of total CoA in the liver of AL (blue diamonds) and CR (red circles) mice (data represented as average±STD, *n* = 4 per time point per diet). (d) Ratio between free and total CoAs in the liver of AL (blue bar) and CR (red bar) mice; *n* = 24 per diet. (e) Daily rhythms of malonyl‐CoA in the liver of AL (blue diamonds) and CR (red circles) mice (data represented as average±STD, *n* = 4 per time point per diet). (f) Representative Western blotting in the liver of AL (marked as a) and CR (marked as c) mice, across the day. Every line represents pooled samples (*n* = 3). (G) Daily rhythms of expression of CPT2 (left panel) and CrAT (right panel) in the liver of AL (blue diamonds) and CR (red circles) mice (data represented as average±STD, *n* = 3 per time point per diet). (h) Ketone bodies (beta‐hydroxybutyrate) in the blood (data represented as average±STD) of AL (*n* = 6) (blue bars), and CR (*n* = 8) (red bars) at ZT14 and ZT18. (i) mRNA expression for *Crat* gene (data represented as average±STD, *n* = 3 per time point per diet). Light and dark bars represent light and dark phases of the day. *—statistically significant difference, *p* < 0.05, *t* test. #—statistically significant effect of diet, *p* < 0.05, two‐way ANOVA. Shadowed area on the graphs or dark bar above the Western blot represents dark phase of the day, the light was on at ZT0, and the light was off at ZT12. Table S7 contains statistics from ANOVA and student's *t* test for acyl‐CoAs

Accumulation of acetylcarnitine is an indication of increased fatty acid oxidation, which agrees with previously reported increases in whole body fat oxidation under CR (Bruss et al., 2010). Beta‐oxidation occurs in mitochondria and peroxisomes. The chain of biochemical reactions is similar between these two organelles, but the enzymes involved in the process are different (Figure 2a). We monitored the expression of enzymes responsible for fatty acid activation, transport to mitochondria and oxidation in the liver by Western blotting. The expression of long‐chain acyl‐CoA synthetase 3 (ACSL3) was slightly induced by CR (Figure 2b and Figure S3). Surprisingly, CR did not affected the expression of CPT1 (Figure 2b and Figure S3), which is considered as one of the key enzymes in mitochondria fat oxidation (Eaton, 2002). CPT1 activity is also regulated by malonyl‐CoA, but malonyl‐CoA level was not affected by CR (Figure 3e). CR induced the expression of both VLCAD and LCAD at multiple times around the clock (Figure 2b,c). The expression of HADHB, a subunit of MTP, that catalyzes the last step of long‐chain acyl‐CoA oxidation was not significantly affected. The expression of MCAD was slightly, but not significantly, upregulated, and the expression of SCAD was not affected by CR. The expression of HADHSC and ACAA2 that catalyze the next steps in oxidation of medium‐ and short‐chain acyl‐CoA was also not significantly affected by CR. (Figure 2b). Thus, the effect of CR on fatty acid metabolic enzyme expression was selective, which changed the balance between different enzymes and contributed to the differential acylcarnitine profile.

Peroxisomes are another site for beta‐oxidation (Figure 2a) (Fransen et al., 2017). ACOX1 is a peroxisomal dehydrogenase and functional analog of ACADs, where it catalyzes the first step of acyl‐CoA beta‐oxidation. In contrast with mitochondrial dehydrogenases, CR did not increase ACOX1 expression and even slightly down regulated the expression (Figure 2b). The expression of peroxisomal thioesterases ACOT3 and/or ACOT4 (the antibodies cannot distinct these two highly homologous enzymes) was increased under CR. ACOTs inactivate acyl‐CoAs by removing the CoA group (Tillander et al., 2017). Thus, the expression of the first committed enzyme in peroxisomal beta‐oxidation was downregulated, and the expression of inactivation enzymes were increased by CR. We concluded that it is unlikely that peroxisomal oxidation of fatty acids was significantly induced under CR, and the majority of increased acylcarnitines are generated in the mitochondria. Peroxisomal beta‐oxidation is not coupled with ATP production; instead, hydrogen peroxide and heat are generated. It is not a surprise that with limited resources under CR, peroxisomal beta‐oxidation is not induced.

To understand the mechanisms of CR‐induced changes in beta‐oxidation enzymes, the mRNA expression for their genes was assayed. The expression of *Acadvl* (encodes VLCAD), *Acadl* (encodes LCAD), and peroxisomal *Acot3* and *Acot4* was significantly induced by CR in a time of the day‐dependent manner (Figure 2d). mRNA expression was in agreement with protein expression and with published data on polysome association (Makwana et al., 2019). Thus, the changes in mRNA expression might provide a mechanistic explanation for increased protein expression.

### 2.3 CR increased accumulation of long‐chain 3‐hydroxy‐acylcarnitines

3‐hydroxy‐acyl‐CoAs are intermediates of beta‐oxidation (Su et al., 2005). Corresponding acylcarnitines are formed by CPT2 (Ramsay, 2000). Several 3‐hydroxy‐acylcarnitines were detected in the liver of AL and CR mice. Daily profiles of 3‐hydroxy‐hexanoylcarnitine (C6‐OH), 3‐hydroxy‐myristoylcarnitine (C14‐OH), and 3‐hydroxy‐octadecenoyl (C18:1‐OH) are shown in Figure 2e, while others are shown in Figure S4A. Most of the detected 3‐hydroxy‐acylcarnitines demonstrated changes through the day in both diets, several of them were rhythmic with 24‐h or 12‐h periods (Figure S4B and Tables S2 and S3). Daily average levels of 3‐hydroxy‐acylcarnitines are presented in Figure S5. There was a statistically significant increase in several long‐chain 3‐hydroxy‐acylcarnitines (C14‐OH, C14:1‐OH, C18‐OH, and C18:1‐OH), and C16:1‐OH demonstrated a tendency to increase (Figure S5C). All detected medium‐chain 3‐hydroxy‐acylcarnitines (C8:1‐OH, C10‐OH, C12‐OH, and C12:1‐OH) were modestly induced by CR (Figure S5B). 3‐hydroxy‐hexanoylcarnitine was reduced by CR in a time of the day‐dependent manner (Figure S5A and Figure 2e). 3‐hydroxybutyrylcarnitine was induced, but the induction was not as strong as the induction in butyrylcarnitine (Figure S5A and Figure 1a). To calculate the ratio between 3‐hydroxy‐acylcarnitine and acylcarnitine, the concentration of 3‐hydroxy‐acylcarnitine in the sample was divided on the concentration of cognate acylcarnitine in the same sample. The calculated ratio across the day is shown in Figure S6. Figure 2f represents the fold change for the ratio between hydroxy‐acylcarnitine and their cognate acylcarnitine (the ratio was set up as 1, shown as dotted line, for every acylcarnitine in AL). CR resulted in several fold increase for long‐chain carnitines (C16:1, C18, C18:1 and C18:2) and a couple medium‐chain carnitines (C10 and C8:1) and several fold decrease for short‐chain carnitines (C4 and C6). Thus, liver 3‐hydroxy‐acylcarnitines were not accumulated in proportion to their cognate acylcarnitine, which further supports significant changes in fatty acid oxidation induced by CR.

### 2.4 CR increases accumulation of short‐chain acyl‐CoA

Acylcarnitines are in dynamic balance with their cognate acyl‐CoAs (Brass & Hoppel, 1980). The increased concentration of short‐chain acylcarnitines is an indication of increased levels of associated acyl‐CoAs. We directly assayed levels of several short acyl‐CoAs and long‐chain palmitoyl‐CoA in the liver. In agreement with acylcarnitine data CR resulted in significant upregulation of acetyl‐CoA, butyryl‐CoA, and hexanoyl‐CoA at all six time points across the day (Figure 3a). Free (Figure 3b) and total (Figure 3c) CoAs were increased by several folds in CR liver suggesting either increased production or reduced degradation of CoA. Interestingly, free and total CoAs were significantly affected by CR but there was no significant difference in free to total ratio between the diets (Figure 3d).

The conversion between acyl‐CoAs and acylcarnitines in the mitochondria is regulated by two mitochondrial carnitine acyltransferases: CPT2 and CrAT. CPT2 acts on long‐chain and medium‐chain acyl‐CoAs (Kerner & Hoppel, 2000), and CrAT acts on short‐chain and medium‐chain acyl‐CoAs including acetyl‐CoA (Seiler et al., 2015). The expression of both enzymes was significantly upregulated by CR (Figure 3f,g). *Crat* mRNA expression was also significantly upregulated in CR liver (Figure 3i). Cytoplasmic short‐chain acyl‐CoA synthases (ACSS2) convert acetate to acetyl‐CoA, and the expression of ACSS2 was not significantly affected by CR (Figure 3f). We also calculated the ratio between acylcarnitines and acyl‐CoA. The results are presented in Figure S7. Interestingly, the ratio between acyl‐CoA and cognate acylcarnitine was changed across the day for both diets and the profiles were different for different acyls. These results suggest that reversible interconversion between acyl‐CoAs and acylcarnitines was not in equilibrium. The changes in CrAT and CPT2 expression might contribute to this dynamic balance.

### 2.5 CR affected blood ketone bodies

Acetyl‐CoA is a precursor for several biochemical reactions, and it can serve as a signaling molecule (Pietrocola et al., 2015). High levels of acetyl‐CoA are frequently associated with increased fat biosynthesis. Malonyl‐CoA is a rate‐limiting precursor for fatty acid biosynthesis and regulator of CPT1 activity. The level of malonyl‐CoA was not increased and even reduced in the CR liver (Figure 3e), which was not surprising. Synthesis of fatty acids from acetyl‐CoA that were produced as a result of fatty acid oxidation would be a futile cycle. Acetyl‐CoA is a precursor for ketone body production (Pietrocola et al., 2015). Blood ketones were assayed at two time points at ZT14 (before feeding CR mice) and at ZT18 (4 h after feeding CR mice). Blood ketone bodies were low at both time points in AL mice (Figure 3h). In CR mice, blood ketone bodies were several folds higher compared with AL at ZT14, and they were as low as AL at ZT18. Interestingly, acetyl‐CoA levels were several folds higher in the CR liver compared with AL at both time points; thus, increased acetyl‐CoA contributed to the increased blood ketones in CR, but additional regulations also exist.

### 2.6 CR increased protein acetylation in the liver

**Figure 4:**
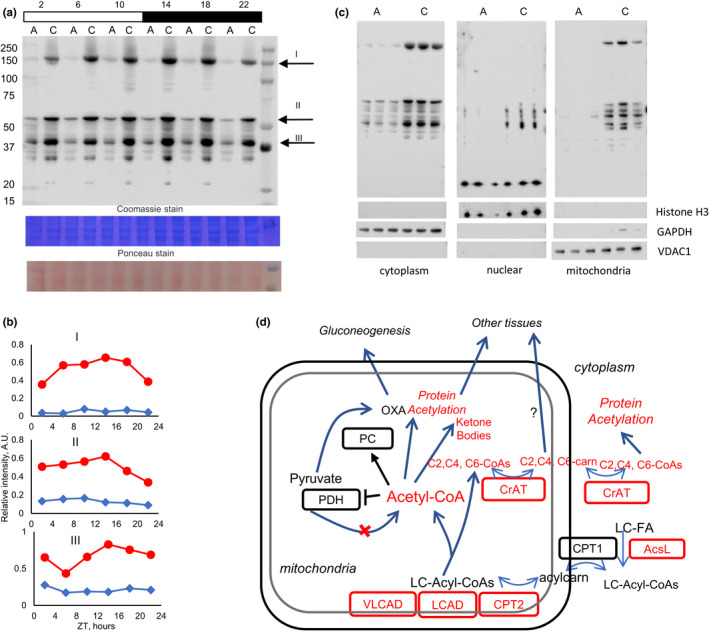
Caloric restriction affected protein acetylation in the liver. (a) Representative Western blotting of protein acetylation in total lysates from the liver of AL (marked as a) and CR (marked as c) mice. (b) Quantification of several major bands from (a), upper panel—band I, middle panel—band II, lower panel—band III. (c) Western blotting of cytoplasmic (left), nuclear (middle), and mitochondrial (right) fractions of the liver, tissues were collected at ZT14. Cellular fraction separation was confirmed by Western blotting for GAPDH (cytoplasm), Histone H3 (nucleus), and VDAC1 (mitochondrial). (d) Effect of CR on acyl‐CoA metabolism (model). After activation and transport to mitochondria long‐chain fatty acids are oxidized by mitochondrial membrane associated VLCAD and VCAD, which expression is increased under CR. As a result, short‐chain acyl‐CoAs are accumulated in the CR liver. Mitochondrial acyl‐CoAs and acylcarnitines are in a dynamic equilibrium controlled by CPT2 and CrAT, which expression is increased by CR. Short‐chain acylcarnitines can travel from mitochondria to cytoplasm, where they can be converted back to acyl‐CoAs. Elevated acetyl‐CoA can be used for ketone body production and for regulation of metabolism, either through the direct suppression of PDH and activation of PC or through protein acetylation. Short‐chain acylcarnitines may also travel into the bloodstream to be used by other tissues for energy production. Components with red text are upregulated under CR

Acetyl‐CoA is a cofactor for protein acetylation, and increased level of acetyl‐CoA correlates with increased protein acetylation. It was previously reported that CR increases protein acetylation in mitochondrial (Schwer et al., 2009) and total (Sato et al., 2017) liver extracts. Protein acetylation in the liver was measured with antibodies to acetyl‐lysine (Figure 4a). Intensity of multiple bands was increased several folds in CR liver across the day. Quantitative data for several bands are shown in Figure 4b. To determine whether increased protein acetylation is exclusively in the mitochondria, liver extracts were fractionated (Figure 4c). The quality of fraction separation was confirmed with known markers histone H3 for the nucleus, GAPDH for the cytoplasm and VDAC1 for the mitochondria. Protein acetylation was measured in cytoplasmic, nuclear, and mitochondrial fractions separately at one time point of the day (ZT14). The increase in acetylation was evident in all fractions. Interestingly, the increase in acetylation was not absolute; for example, there was no increase in the intensity of 17‐kDa band in nuclear fractions. Protein acetylation can be catalyzed by protein acetylases, or it can be enzyme‐independent (Pietrocola et al., 2015), and an increase in acetyl‐CoA shall stimulate both. At the same time, protein deacetylases have substrate preferences, and observed differences in acetylation pattern might be due to the regulatory change of protein deacetylase activity, which warrants further study.

### 2.7 CR differentially affected expression of PPARα transcriptional targets

PPARα is a transcriptional factor and master regulator of liver fat metabolism (Kersten et al., 1999). It was hypothesized that PPARα activity might be induced by CR (Masternak & Bartke, 2007), but it was never directly demonstrated. Several of the upregulated fat oxidation enzymes are known targets of PPARα; therefore, we decided to investigate whether several other PPARα targets were affected in CR liver. The expression for all assayed PPARα transcriptional targets was significantly affected in CR liver (Figure 2d, 3i and Figure S8). *Acadl*, *Acadvl*, *Crat*, *Hadhb*,*Slc25a20*,*Acot3*, and *Acot4* were significantly induced at several time points. *Acadm* and *Cpt2* were induced only at one time point. Diet had a significant effect on *Acads*, *Cpt1a*, and *Hmgcs2* expression, but the difference did not reach significance at any time point (see details of statistical analysis in Table S4). A ketogenic diet is a popular dietary intervention to improve metabolism, and there are some parallels between CR and ketogenic diet. Recently, an analysis of the liver transcriptome around the clock in mice on ketogenic diet concluded that PPARα transcriptional activity is induced by ketogenic diet (Tognini et al., 2017). We analyzed available RNAseq data from Tognini et al and compared it with the expression in our study. The expression of these PPARα targets is strongly upregulated by ketogenic diet at all time points around the clock, which is in contrast with CR that have a time of the day‐dependent effect and overall minimal induction (Tognini et al., 2017). Protein expression was not reported; therefore, it is unclear if ketogenic diet induced mRNA expression will translate to the protein level. Thus, CR and ketogenic diet have significantly different effects on the expression of fat oxidation enzymes and PPARα transcriptional targets.

## 3 DISCUSSION

Fatty acid beta‐oxidation is the main mechanism of energy production under CR, and a strong flux of fatty acids to the liver is expected under CR (Anderson & Weindruch, 2010; Bruss et al., 2010). Profiling of acylcarnitines is a widely accepted method to detect changes in fatty acid oxidation (El‐Gharbawy & Vockley, 2018). We performed a comprehensive circadian analysis of acylcarnitines and unexpectedly found a selective effect of CR on acylcarnitine and acyl‐CoA rhythms in the liver. Acetylcarnitine and acetyl‐CoA levels were previously assayed in CR with mixed results. Acetylcarnitine is elevated by acute CR in human serum (Collet et al., 2017), which agrees with our data. At the same time, reduced acetyl‐CoA was observed by (Hebert et al., 2013) and (Perry et al., 2018). Finally, Gopalan et al reported no significant effect on acetylcarnitine levels (Gopalan et al., 2016). We do not know what exactly causes the difference between our study and cited studies: Hebert et al performed analysis only in one sample, Perry et al used a diabetic rat model and severe 4‐day CR, Gopalan et al used high‐fat diet induced obesity rat model and CR that was implemented with high‐fat diet. Methods of acetyl‐CoA analysis were different between studies. Data on protein acetylation under CR from our study and two other studies (Sato et al., 2017; Schwer et al., 2009) are in line with the increase in acetyl‐CoA in CR liver. Gopalan et al also reported an induction for a few long‐chain acylcarnitines; however, the effect was significant for 15% CR but not 30% CR (Gopalan et al., 2016). To the best of our knowledge, the effect of CR on acylcarnitine profile in healthy animals has not been reported.

The expression of several fat oxidation genes is affected by CR according to several transcriptomic studies (Swindell, 2009), but protein expression was not reported. The expression of CPT1 and its physiological regulator malonyl‐CoA were not significantly affected by CR. CR significantly increased the expression of VLCAD and LCAD, which are enzymes involved in the initial steps of fatty acid beta‐oxidation, but did not increase the expression of downstream enzymes such as trifunctional protein subunits or any of the enzymes involved in medium and short‐chain beta‐oxidation. These results are in contrast with the effect of ketogenic diet which results in significant upregulation of mRNAs for all fat oxidation enzymes (Tognini et al., 2017). Of note, only mRNA data are available from Tognini et al, and the effect of ketogenic diet on protein expression was not reported. CR and ketogenic induced changes in acylcarnitine profiles were also different. Medium and long‐chain, but not short‐chain acylcarnitines, accumulate in the liver of mice after 4 days on ketogenic diet (Lee et al., 2016). Thus, CR and ketogenic diet differently reprogram fat metabolism. CR did not simply increase fat oxidation; rather, the balance between upstream and downstream enzymes was significantly shifted, which in turn might contribute to the observed changes in the acylcarnitine profile.

We also compared the acylcarnitine profiles in CR liver with acylcarnitine profiles in tissues and body fluids from other studies using animal models and clinical cases of fatty acid oxidation deficiency. SCAD deficient mice have increased hexanoylcarnitine in the liver, but butyrylcarnitine and acetylcarnitine are not increased, and medium and long‐chain acylcarnitines, which are upstream in beta‐oxidation pathways, are also significantly upregulated (Ghosh et al., 2016). MCAD deficient mice have increased accumulation of C6‐C10 acylcarnitines and no increase in short‐chain acylcarnitines (Tolwani et al., 2005). LCAD deficient mice have almost opposite acylcarnitine profile to the profile of CR: increased long‐chain acylcarnitines, reduced level of 3‐hydroxy‐acylcarnitines (Kurtz et al., 1998; Van Vlies et al., 2005), and no accumulation of short‐chain acylcarnitines. Trifunctional protein deficiency results in accumulation of both long‐chain hydroxy‐acylcarnitine and long‐chain acylcarnitines, but there is no accumulation of short‐chain acylcarnitines (Van Hove et al., 2000). Importantly, in each discussed fatty acid oxidation deficiency disorder the level of acetylcarnitine is not increased, which agrees with reduced beta‐oxidation (Ghosh et al., 2016). Thus, while short‐chain acylcarnitines were accumulated in CR, the profile is significantly different from fat oxidation deficiency.

Increased acetyl‐CoA suggests that production did not match consumption. Acetyl‐CoA can be consumed through TCA cycle, ketone body production or exported from mitochondria either as citrate or as acetylcarnitine. Other short‐chain acyl‐CoAs can also be exported as cognate carnitines. We do not know if acetyl‐CoA through TCA cycle was affected by CR. Blood ketones were induced, at least at ZT14, in agreement with increased acetyl‐CoA. Protein acetylation in different cellular compartments was increased, implicating acetyl‐CoA export from the mitochondria. One export pathway relies on the generation of acetylcarnitine by mitochondrial CrAT and export via the carnitine shuttle. After export from the mitochondria, acetylcarnitine is converted back to acetyl‐CoA by cytoplasmic CrAT (Altamimi et al., 2018; Seiler et al., 2015). There was a significant induction in the expression of CrAT under CR conditions. High level of acetylcarnitine and increased expression of CrAT suggest that the carnitine shuttle pathway of export was induced in CR. Another pathway for acetyl‐CoA export from mitochondria is “citrate–malate–pyruvate shuttle.” It is known that citrate transport plays an important role in fat biosynthesis when there is high carbohydrate load. It is hypothesized that this pathway is reduced in fasting due to depletion of oxaloacetate for gluconeogenesis (Pietrocola et al., 2015). It is unknown if the citrate shuttle is affected in CR. Therefore, we cannot exclude that citrate transport contributes to acetyl‐CoA export. Finally, it was suggested that increased acetate is responsible for increased acetyl‐CoA in CR liver (Sato et al., 2017), but it was not experimentally demonstrated.

What might be a physiological significance for the increase of short‐chain acyl‐CoAs, acetyl‐CoAs and corresponding acylcarnitines? What might be their roles in CR health benefits? Based on our data, we proposed a following model on CR‐induced changes in fat oxidation and short‐chain acyl‐CoA homeostasis and how it might impact metabolism and physiology (Figure 4d).

1. Acetyl‐CoA and other short‐chain acyl‐CoAs can be used for protein posttranslational modifications. Our data expanded the results of several previous reports on CR and protein acetylation. CR induces protein acetylation in the mitochondria (Schwer et al., 2009), while the effect might be protein specific (Hebert et al., 2013), and total (Sato et al., 2017) liver extracts. Our results suggest that acetylation of cytoplasmic and nuclear proteins is also induced in the liver by CR (Figure 4c). In agreement with our data, 24‐h fasting results in increased protein acetylation in the liver of wild‐type mice but not in the liver of mice deficient for LCAD (Pougovkina et al., 2014). Thus, the activity of long‐chain acyl‐CoA dehydrogenases is necessary for increased acetyl‐CoA production during fasting and, probably, under CR, which needs to be tested in the future.
2. Fatty acids are natural ligands for transcriptional factors from the PPAR family, including PPARα and different ligands affect different subsets of targets (Kersten et al., 1999). Here, we found that CR differentially regulated the expression of several canonical PPARα targets (Mandard et al., 2004) in the liver. Changes in the acyl profile induced by CR might modulate PPARα activity and contribute to changes in the transcriptional landscape under CR. It might also affect a crosstalk with other transcriptional regulators, such as circadian clock transcriptional factors (Eckel‐Mahan et al., 2013; Mukherji et al., 2015).
3. Finally, acylcarnitines can travel in the blood stream, and they can be released or consumed by tissues depending on energy demands. For example, skeletal muscles efflux acetylcarnitine at rest and uptake acetylcarnitine during the physical activity (Seiler et al., 2015; Simcox et al., 2017). Changes in acylcarnitine flux might contribute to metabolic flexibility of the tissues during nutrient and energy stress (Muoio, 2014). Therefore, under CR conditions the liver may provide energy supply to other tissues in the form of short‐chain acylcarnitines.
4. Finally, acetyl‐CoA and other short‐chain acyl‐CoAs may also act as signaling and regulatory molecules. High levels of short‐chain acyl‐CoAs are associated with reduced glycolysis and increased gluconeogenesis. For example, pyruvate dehydrogenase activity is suppressed, and pyruvate carboxylase activity is stimulated by acetyl‐CoAs, thus, redirecting pyruvate from TCA cycle to gluconeogenesis, which is important during prolonged fasting in CR.

Limitations of the study: acetyl‐CoA is generated as a result of various catabolic processes. While our data strongly implicate that beta‐oxidation is the main contributor to the increase in acetyl‐CoA, it needs to be directly tested. Similarly, it was not directly assayed if increased butyryl‐CoA and hexanoyl‐CoA were produced through the oxidation of stored triglycerides or from gut microbiota derived short‐chain fatty acids. Muscle and adipose tissue play an important role in whole body metabolism, but this study was limited to the liver. There is evidence that the outcome might be different in different tissues. Indeed, CR increases protein acetylation in the liver mitochondria, but in several other tissues the effect is minimal if any (Schwer et al., 2009). Changes in protein acetylation may be due to changes in the activities of protein deacetylases and acetylases, which were not assayed. At the same time, it is expected that activities of deacetylases will be increased under CR, and activities of, at least some, acetylases will be decreased due to increased blood ketones.

## 4 EXPERIMENTAL PROCEDURE

Detailed methods and list of reagents are available in Appendix S1.

### 4.1 Animals

All experiments involving animals were conducted in accordance with Federal and University guidelines, and all procedures were approved by IACUC, Cleveland State University. C57BL/6J mice were maintained on 12 h light: 12 h dark cycle (LD12:12). Mice used in the experiments were 12–13 weeks of age at the start of the experiments. Animals were maintained in groups of three–four animals per cage on 5008 LabDiet (proteins 26.5%, fat 16.9%, carbohydrates 56.5%). CR mice received food once per day 2 h after the lights were turned off (ZT14). At 5 months of age, liver tissues were collected across the day, every 4 h and immediately frozen and kept at −80°C for subsequent processing.

### 4.2 Blood Glucose and β‐hydroxybutyrate detection

Blood for the analysis was collected through the tail vein at time points ZT2, 6, 10, 14, 18, and 22. Blood ketones were measured as β‐hydroxybutyrate level using Precision Xtra Blood Glucose and Ketone meter (Abbott Laboratories, IL, USA).

### 4.3 Analysis of mRNA expression

Total RNA was extracted from frozen liver tissue using TRIzol according to manufacturer's instructions. After quantification and quality check of total RNA by electrophoresis, total RNA was reverse transcribed with SuperScript IV Reverse Transcriptase. Real‐time quantitative PCR was performed using iTaq Universal SYBR Green Supermix (Bio‐Rad). 18S rRNA expression levels were used for normalization. Fold change was determined by ΔΔCt method.

### 4.4 Analysis of protein expression

Total liver lysates were prepared with lysis buffer (1 M Tris Base pH 7.5, 5 M NaCl, 0.5 M EGTA, 0.5 M EDTA, Triton‐X, 0.1 M Na_4_P_2_O_7_, 1 M β‐glycerophosphate, 1 M Na_3_VO_4_) containing protease and phosphatase inhibitor cocktails (Sigma). For fractionated protein extracts, 30 mg of liver was homogenized with Dounce homogenizer in Buffer A (10 mM HEPES, 1.5 mM MgCl2, 10 mM KCl, 0.5 mM DTT, 0.05% NP40, 0.1 M Na4P2O7, 1 M β‐glycerophosphate, 1 M Na3VO4, and protease and phosphatase inhibitor cocktails) and centrifugated at 960 *g* for 5 min at rcf: 4°C. The pellet contained the nuclear fraction, and supernatant was centrifugated at rcf: 6800 *g* for 5 min for separation of cytosolic (supernatant) and mitochondria (pellet) fractions. After SDS‐PAGE, proteins were transferred onto PVDF membrane. For list of antibodies, see Supplemental Key Resources Table. Quantification of images was done using Image Studio Lite software.

### 4.5 Sample Preparation for Acylcarnitine and acyl‐CoA Analysis

Internal standard stock solutions were prepared as follows: Acylcarnitine Internal Standard Mix (1520 nM d9‐carnitine, 380 nM d3‐acetylcarnitine, 76 nM d3‐propionylcarnitine, 76 nM d3‐butyrylcarnitine, 76 nM d9‐isovalerylcarnitine, 76 nM d3‐octanoylcarnitine, 76 nM d9‐myristoylcarnitine, 152 nM d3‐palmitoylcarnitine) in methanol. 13C_2_‐acetyl‐CoA (1000 ng/ml in 1:1 v/v methanol:5% acetic acid).

All sample preparations were performed on ice. Mouse liver tissues (~40 mg frozen mass stored at −80°C) were spiked with 10 µl of NSK‐B acylcarnitine internal standard mix (Cambridge Isotope Labs) and 10 µl of 1000 ng/ml 13C_2_‐acetyl‐CoA (Sigma Aldrich). Each tissue was homogenized in 1 ml of “Extraction Matrix” (1:1 v/v) methanol:5% acetic acid (aq) using a VWR pellet mixer and centrifuged at 12 k rpm for 5 min. The supernatant was transferred to a 13 mm glass tube. The remaining tissue was homogenized and centrifuged again in 1 ml of Extraction Matrix. The combined supernatants were dried under N_2_ and reconstituted in 200 µl of Extraction Matrix. A total of 50 µl was aliquoted for acylcarnitine analysis, and the remaining kept at −80°C for acyl‐CoA analysis. The 50 µl aliquot was dried under N_2_ and derivatized by addition of 60 µl butanol‐1‐HCl (Sigma Aldrich) incubated at 80°C for 20 min. The sample was dried under N_2_ and reconstituted in 60 µl of (80:20:0.1 v/v/v) acetonitrile:water:formic acid. The samples were stored at −80°C. For analysis, frozen samples were kept on ice and transferred to HPLC vials with 100 µl glass inserts after vortex mixing.

### 4.6 Acylcarnitine Analysis by LC‐MS/MS

Analysis was performed using a Shimadzu Nexera UHPLC coupled to a SCIEX QTrap 5500 mass spectrometer. The SCIEX Turbo V ESI source was operated in positive mode with curtain gas set to 40 psi, CAD gas set to high, IS voltage 4500 V, temperature 700°C, GS1 and GS2 gases at 50 psi and 45 psi, respectively. Compound‐specific parameters were optimized by direct infusion of a 1 µM mixture of butylated free carnitine, acetylcarnitine, butyrylcarnitine, octanoylcarnitine, and palmitoylcarnitine in 0.1% formic acid in acetonitrile. Butylated acylcarnitines fragment similarly producing a common fragment m/z 85 in positive mode. Therefore, the MRM transitions for acylcarnitines of interest were measured by monitoring the parent ion [M + H]+ > m/z 85.^1,2^ Separation was achieved using a Waters XBridge BEH C18 (75 × 2.1 mm, 2.5 μm) column heated to 40°C with flow rate 0.6 ml/min. The autosampler was kept at 4°C and sample injection volume set to 1 µl. The needle was rinsed externally after each injection with acetonitrile. Gradient elution: 0.1% formic acid in HPLC‐grade water (mobile phase A) and 0.1% formic acid in acetonitrile (mobile phase B). Time program: 1 min hold at 20% B, linear increase to 37% B at 8 min, and linear increase to 100% B at 22 min. Hold at 100% B for 4 min followed by re‐equilibration at starting conditions for 4 min. Total run time was 30 min per sample.

### 4.7 Acyl‐CoA Analysis by LC‐MS/MS

Analysis was performed using a Shimadzu Nexera UHPLC coupled to a SCIEX QTrap 5500 mass spectrometer. The SCIEX Turbo V ESI source was operated in positive mode with curtain gas set to 30 psi, CAD gas set to medium, IS voltage 5000 V, temperature 550°C, GS1 and GS2 gases at 40 psi. Compound‐specific parameters were optimized by direct infusion of a 1000 ng/ml mixture of free CoA, acetyl‐CoA, succinyl‐CoA, glutaryl‐CoA, acetoacetyl‐CoA, propionyl‐CoA, octanoyl‐CoA, palmitoyl‐CoA, octadecenoyl‐CoA, and 3‐hydroxybutyryl‐CoA. Acyl‐CoAs produce the common fragment m/z 507. The acyl‐CoAs of interest were detected by monitoring the MRM transition of parent ion [M + H]+ to the appropriate fragment ion produced by the loss of m/z 507.^3,4^ Separation was achieved using a Waters Acquity CSH C18 (50 × 2.1 mm, 1.7 μm) column heated to 30°C with flow rate 0.6 ml/min. The autosampler was kept at 4°C and sample injection volume set to 5 µl. The needle was rinsed externally after each injection with (3:2:1:0.2 v/v/v/v) acetonitrile:isopropanol:water:phosphoric acid. Gradient elution: 100 mM ammonium formate in 98:2 v/v water:acetonitrile (mobile phase A) and 5 mM ammonium formate in 95:5 v/v acetonitrile:water. (mobile phase B). Time program: 1 min hold at 2.5% B, linear increase to 55% B at 4 min, and linear increase to 98% B at 6 min. Hold at 98% B for 2 min followed by re‐equilibration at starting conditions for 7 min. Total run time was 15 min per sample.

### 4.8 Quantification and Statistical Analysis

Data for both diets were analyzed using ordinary two‐way ANOVA (multiple comparison correction was done using the Bonferroni method). All statistical analysis was performed using GraphPad Prism 7.0 (San Diego, CA). Rhythms analysis was performed in R‐studio using MetaCycle package (Wu et al., 2016). At least three biological replicates have been used for every time point and every diet. Exact number of biological samples for every experiment is reported in the Figure legends. Data are shown as Mean ± SD. *p* ≤ 0.05 was considered as statistically significant difference.

## CONFLICT OF INTEREST

The authors declare no competing interests.

## AUTHOR CONTRIBUTIONS

V.M., R.P., Y.S., and R.K. conceived and designed the study. V.M., R.P., A.P., N.V., A.A., K.M., and O.E. performed experiments. V.M., R.P., A.P., Y.S., and R.K. analyzed the data. V.M. and R.K. prepared the figures and wrote the manuscript. All authors contributed to reading, revision, and approval of the manuscript.

## Supporting information

Appendix S1

## Data Availability

The data that support the findings of this study are openly available in Mendeley Data at V2, https://doi.org/10.17632/9f34sdmkf2.2.
