# Supplementary material for: CR reprograms acetyl‐CoA metabolism and induces long‐chain acyl‐CoA dehydrogenase and CrAT expression
Source: Aging Cell. 2020 Oct 26;19(11):e13266. doi: 10.1111/acel.13266 (PMC7681051; doi:10.1111/acel.13266)
Supplement: Supplementary file 1 — Appendix S1 [file ACEL-19-e13266-s001.docx]

**Supplementary Figures**

**Figure S1.** (related to Figure 1). Daily average of acylcarnitines (absolute values) in the liver of AL (blue bars) and CR (red bars) mice (Data represented as average+/- STD, n=24 per diet). (A) Short-chain, (B) medium-chain, (C) long-chain, (D) free carnitines and (E) total acylcarnitines. Acylcarnitine species are annotated as following [number of carbon atoms]:[number of double bonds]. * - statistically significant difference, p<0.05. Supplementary Table 1 contains statistics from anova and student’s T-test for acylcarnitines.

**Figure S2.** (related to Figure 1). (A) Daily rhythms of acylcarnitines in the liver of AL (blue lines) and CR (red lines) mice (Data represented as average+/- STD, n=4 per time point per diet). (B) Phase distribution of rhythmic acylcarnitines with 12-hour and 24-hour periods as indicated, AL (blue) and CR (red). # - statistically significant effect of diet, p<0.05, two-way ANOVA. Shadowed area on the graphs represents dark phase of the day, the light was on at ZT0 and the light was off at ZT12. Supplementary Table 2 and 3 contains statistics for 24-hour rhythm 12-hour rhythm analysis, respectively.

**Figure S3.** (related to Figure 2). Protein expression of beta-oxidation enzymes in the liver of AL (blue diamonds) and CR (red circles) mice (Data represented as average+/- STD, n=3 per time point per diet). Shadowed area on the graphs represents dark phase of the day, the light was on at ZT0 and the light was off at ZT12. Supplementary Tables 4 and 5 contain statistics from anova and student’s T-test for qPCR and protein results, respectively.

**Figure S4.** (related to Figure 2). (A) Daily rhythms of hydroxy-acylcarnitines in the liver of AL (blue lines) and CR (red lines) mice (Data represented as average+/- STD, n=4 per time point per diet). (B) Phase distribution of rhythmic 3-hydroxy-acylcarnitines with 12-hour and 24-hour periods as indicated, AL (blue) and CR (red). # - statistically significant effect of diet, p<0.05, two-way ANOVA. Shadowed area on the graphs represents dark phase of the day, the light was on at ZT0 and the light was off at ZT12. Supplementary Table 6 contain statistics from anova and student’s T-test for hydroxy-acylcarnitines quantifications.

**Figure S5.** (related to Figure 2). Daily average of hydroxy-acylcarnitines (absolute values) in the liver of AL (blue bars) and CR (red bars) mice (Data represented as average+/- STD, n=24 per diet). (A) Short-chain, (B) medium-chain, (C) long-chain hydroxy-acylcarnitines. Acylcarnitine species are annotated as following [number of carbon atoms]:[number of double bonds]. * - statistically significant difference, p<0.05.

**Figure S6.** (related to Figure 2). Daily rhythms in ratio between 3-hydroxy-acylcarnitines and corresponding acylcarnitines in the liver of AL (blue lines) and CR (red lines) mice. Shadowed area on the graphs represents dark phase of the day, the light was on at ZT0 and the light was off at ZT12.

**Figure S7.** (related to Figure 3). Daily rhythms in ratio between acyl-CoAa and their corresponding acylcarnitines in the liver of AL (blue lines) and CR (red lines) mice. To calculate the ration average values for acyl-CoAs at every time point were divided by average values for corresponding acylcarnitine. Shadowed area on the graphs represents dark phase of the day, the light was on at ZT0 and the light was off at ZT12. Supplementary Table 7 contains statistics from anova and student’s T-test for acyl-CoAs.

**Figure S8.** Effect of CR on daily rhythms in the expression of selected PPARa transcriptional targets. mRNA expression for indicated genes (Data represented as average+/- STD, n=3 per time point per diet) in the liver of AL (blue lines) and CR (red lines) mice. # - statistically significant effect of diet, p<0.05, two-way ANOVA. Shadowed area on the graphs represents dark phase of the day, the light was on at ZT0 and the light was off at ZT12.

**Supplementary Tables**

Supplementary Table 1. Statistical analysis for acylcarnitines.

| **ANOVA (Diet)** | | | | | **p Value; Multiple Comparisons (Time)** | | | | | |
| --- | --- | --- | --- | --- | --- | --- | --- | --- | --- | --- |
| **Acylcarnitine** | **Mean Amount (nmol / g tissue)** | | **Standard Deviation** | **P value** | **2** | **6** | **10** | **14** | **18** | **22** |
|  |  |  |  |  |  |  |  |  |  |  |
| C0 | AL | 208.00 | 51.96 | 0.020 | 0.04 | 0.27 | 0.64 | 0.86 | 0.02 | >0.99 |
|  | CR | 364.20 | 151.90 |  |  |  |  |  |  |  |
| C2 | AL | 24.39 | 6.67 | <0.001 | 0.04 | 0.01 | >0.99 | 0.05 | 0.02 | 0.00 |
|  | CR | 507.50 | 141.10 |  |  |  |  |  |  |  |
| C4 | AL | 4.61 | 1.83 | <0.001 | 0.06 | 0.39 | >0.99 | 0.06 | 0.11 | 0.06 |
|  | CR | 67.55 | 40.15 |  |  |  |  |  |  |  |
| C6 | AL | 2.44 | 2.63 | 0.040 | >0.99 | 0.81 | >0.99 | >0.99 | >0.99 | >0.99 |
|  | CR | 8.98 | 8.43 |  |  |  |  |  |  |  |
| C8 | AL | 1.01 | 0.49 | 0.540 | >0.99 | 0.35 | >0.99 | >0.99 | >0.99 | 0.79 |
|  | CR | 0.89 | 0.47 |  |  |  |  |  |  |  |
| C10 | AL | 1.14 | 0.61 | 0.430 | >0.99 | 0.73 | >0.99 | >0.99 | 0.28 | >0.99 |
|  | CR | 1.02 | 0.49 |  |  |  |  |  |  |  |
| C12 | AL | 0.39 | 0.22 | 0.030 | >0.99 | 0.07 | >0.99 | >0.99 | 0.69 | >0.99 |
|  | CR | 0.92 | 0.46 |  |  |  |  |  |  |  |
| C14 | AL | 1.23 | 0.83 | 0.008 | 0.26 | 0.13 | >0.99 | 0.51 | >0.99 | 0.28 |
|  | CR | 2.57 | 1.22 |  |  |  |  |  |  |  |
| C16 | AL | 8.79 | 2.94 | 0.710 | >0.99 | 0.66 | >0.99 | >0.99 | 0.51 | >0.99 |
|  | CR | 9.13 | 2.62 |  |  |  |  |  |  |  |
| C18 | AL | 1.37 | 0.42 | 0.030 | 0.33 | >0.99 | 0.30 | >0.99 | >0.99 | >0.99 |
|  | CR | 1.10 | 0.32 |  |  |  |  |  |  |  |
| C12:1 | AL | 0.58 | 0.35 | 0.370 | >0.99 | 0.37 | >0.99 | >0.99 | 0.09 | >0.99 |
|  | CR | 0.76 | 0.44 |  |  |  |  |  |  |  |
| C14:1 | AL | 0.76 | 0.48 | 0.001 | 0.06 | <0.001 | >0.99 | 0.47 | 0.80 | 0.01 |
|  | CR | 3.71 | 1.66 |  |  |  |  |  |  |  |
| C16:1 | AL | 3.26 | 2.61 | 0.320 | >0.99 | >0.99 | 0.81 | >0.99 | >0.99 | >0.99 |
|  | CR | 2.85 | 1.62 |  |  |  |  |  |  |  |
| C16:2 | AL | 0.59 | 0.16 | 0.002 | <0.001 | >0.99 | >0.99 | >0.99 | >0.99 | >0.99 |
|  | CR | 1.06 | 0.55 |  |  |  |  |  |  |  |
| C18:1 | AL | 4.79 | 1.62 | 0.810 | >0.99 | 0.26 | >0.99 | >0.99 | >0.99 | >0.99 |
|  | CR | 5.00 | 1.92 |  |  |  |  |  |  |  |
| C18:2 | AL | 10.24 | 4.26 | 0.006 | 0.84 | >0.99 | 0.31 | >0.99 | 0.68 | 0.10 |
|  | CR | 4.32 | 1.28 |  |  |  |  |  |  |  |
|  | CR | 0.35 | 0.28 |  |  |  |  |  |  |  |

Supplementary Table 2. 24-hour rhythms of liver acylcarnitines.

| **Acylcarnitine** | **AL phase** | **CR phase** | **AL p-value** | **CR p-value** |
| --- | --- | --- | --- | --- |
| C0 | No | 20 | 0.491149681 | 0.000339769 |
| C2 | No | No | 0.548070549 | 0.439008008 |
| C4 | No | 0 | 0.913441549 | 0.025449507 |
| C4-OH | No | No | 0.272901515 | 1 |
| C6 | No | 22 | 1 | 0.030246094 |
| C6-OH | No | No | 0.132260786 | 1 |
| C8 | No | No | 1 | 0.211661635 |
| C8:1 | No | No | 1 | 1 |
| C8:1-OH | No | 8 | 1 | 0.006846141 |
| C10 | No | 6 | 1 | 0.042276068 |
| C10-OH | No | No | 0.677305858 | 0.548070549 |
| C10:1 | No | No | 1 | 0.12301136 |
| C10:2 | No | 6 | 0.828769317 | 8.95E-06 |
| C10:3 | No | No | 0.913441549 | 1 |
| C12 | No | No | 1 | 0.677305858 |
| C12-OH | No | No | 1 | 0.058294166 |
| C12:1 | No | 6 | 0.548070549 | 0.017823892 |
| C12:1-OH | No | 8 | 0.141510212 | 0.010157419 |
| C14 | 18 | 20 | 0.042276068 | 0.048294166 |
| C14-OH | No | No | 1 | 1 |
| C14:1 | No | No | 1 | 1 |
| C14:1-OH | 8 | No | 0.014833899 | 0.828769317 |
| C14:2 | No | No | 0.610035673 | 0.677305858 |
| C16 | No | No | 1 | 1 |
| C16-OH | 16 | No | 0.021337014 | 1 |
| C16:1 | No | No | 1 | 1 |
| C16:1-OH | No | No | 1 | 0.913441549 |
| C16:2 | No | No | 0.491149681 | 1 |
| C18 | No | No | 1 | 1 |
| C18-OH | No | 16 | 0.677305858 | 0.025449507 |
| C18:1 | No | No | 1 | 0.185616664 |
| C18:1-OH | No | No | 0.09210924 | 1 |
| C18:2 | No | No | 1 | 0.491149681 |
| C18:2-OH | 14 | No | 0.021337014 | 1 |
| C20-OH | No | No | 0.185616664 | 0.240675842 |
| C20:4 | No | No | 1 | 0.677305858 |

Supplementary Table 3. 12-hour rhythms of liver acylcarnitines.

| **Acylcarnitine** | **AL phase** | **CR phase** | **AL p-value** | **CR p-value** |
| --- | --- | --- | --- | --- |
| C0 | 0 | No | 0.021138034 | 0.717213386 |
| C2 | No | No | 1 | 0.602899188 |
| C4 | No | No | 0.779633908 | 0.136450758 |
| C4-OH | No | 8 | 0.136450758 | 0.021138034 |
| C6 | No | No | 0.24557484 | 0.375067664 |
| C6-OH | No | No | 0.68776683 | 1 |
| C8 | 10 | No | 0.04605462 | 0.338652929 |
| C8:1 | No | No | 0.136450758 | 0.259805057 |
| C8:1-OH | No | No | 0.092808332 | 0.915204992 |
| C10 | 10 | No | 0.04605462 | 0.915204992 |
| C10-OH | No | No | 0.845622691 | 0.274035274 |
| C10:1 | No | No | 0.988389501 | 1 |
| C10:2 | No | No | 0.414384659 | 1 |
| C10:3 | No | No | 0.375067664 | 1 |
| C12 | 10 | No | 0.04605462 | 0.414384659 |
| C12-OH | No | No | 1 | 0.915204992 |
| C12:1 | No | No | 0.414384659 | 0.988389501 |
| C12:1-OH | No | No | 1 | 1 |
| C14 | No | No | 0.305017837 | 0.915204992 |
| C14-OH | 8 | 2 | 0.024863017 | 0.010668507 |
| C14:1 | 0 | 2 | 0.015123047 | 0.024863017 |
| C14:1-OH | No | 2 | 0.375067664 | 0.008911946 |
| C14:2 | No | No | 0.779633908 | 0.105830818 |
| C16 | 0 | No | 0.012724754 | 0.915204992 |
| C16-OH | No | No | 0.195689199 | 0.120337921 |
| C16:1 | No | No | 0.057404891 | 0.356860297 |
| C16:1-OH | No | 2 | 0.602899188 | 0.001478892 |
| C16:2 | No | 2 | 1 | 0.015123047 |
| C18 | 0 | No | 0.001836046 | 0.658320274 |
| C18-OH | 6 | No | 0.034057521 | 1 |
| C18:1 | No | No | 0.053304103 | 0.305017837 |
| C18:1-OH | No | 2 | 0.219504004 | 0.008911946 |
| C18:2 | 10 | No | 0.024863017 | 0.053304103 |
| C18:2-OH | No | 2 | 0.053304103 | 0.04605462 |
| C20-OH | No | No | 0.658320274 | 1 |
| C20:4 | 0 | 4 | 0.002269433 | 0.012724754 |

Supplementary Table 4. Statistics for qPCR analysis.

| **ANOVA (Diet)** | | | | **p Value; Multiple Comparisons (Time)** | | | | | | | |  |  |
| --- | --- | --- | --- | --- | --- | --- | --- | --- | --- | --- | --- | --- | --- |
| **Gene** | | **P value** | | **2** | | **6** | **10** | **14** | **18** | **22** | |  |  |
| *Acadm* | | < 0.0001 | | > 0.9999 | | 0.1657 | 0.0068 | 0.3569 | 0.5808 | > 0.9999 | |  |  |
| Acads | | 0.0302 | | > 0.9999 | | > 0.9999 | 0.3205 | > 0.9999 | 0.619 | > 0.9999 | |  |  |
| *Cpt2* | | 0.0013 | | 0.3404 | | > 0.9999 | > 0.9999 | > 0.9999 | 0.0929 | 0.0022 | |  |  |
| *Cpt1a* | | 0.0165 | | > 0.9999 | | > 0.9999 | 0.2685 | 0.297 | > 0.9999 | > 0.9999 | |  |  |
| *Acot3* | | < 0.0001 | | 0.0001 | | 0.6782 | 0.2969 | < 0.0001 | 0.4592 | > 0.9999 | |  |  |
| *Acot4* | | < 0.0001 | | > 0.9999 | | > 0.9999 | 0.0879 | < 0.0001 | 0.0043 | 0.145 | |  |  |
| *Slc25a20* | | < 0.0001 | | > 0.9999 | | 0.2589 | 0.028 | > 0.9999 | 0.0088 | > 0.9999 | |  |  |
| *Acadvl* | | < 0.0001 | | 0.0017 | | > 0.9999 | 0.0024 | 0.0075 | 0.0745 | 0.2669 | |  |  |
| *Acadl* | | < 0.0001 | | 0.0007 | | > 0.9999 | 0.0007 | 0.0081 | < 0.0001 | 0.1893 | |  |  |
| *Hadha* | | 0.0004 | | 0.818 | | > 0.9999 | 0.0291 | > 0.9999 | 0.0548 | 0.5752 | |  |  |
| *Crat* | | < 0.0001 | | > 0.9999 | | > 0.9999 | 0.2201 | 0.0036 | 0.03 | 0.0987 | |  |  |
| *Hmgcs2* | | 0.0135 | | 0.2971 | | > 0.9999 | > 0.9999 | > 0.9999 | > 0.9999 | 0.8288 | |  |  |
|  | |  | |  | |  |  |  |  |  | |  |  |
|  |  | |  | |  | | | | | |  | |  |

Supplementary Table 5. Statistics for WB analysis.

| **ANOVA (Diet)** | | **p Value; Multiple Comparisons (Time)** | | | | | |
| --- | --- | --- | --- | --- | --- | --- | --- |
| **Gene** | **P value** | **2** | **6** | **10** | **14** | **18** | **22** |
| LCAD | <.0001 | 0.04 | 0.02 | 0.007 | 0.25 | 0.02 | 0.0008 |
| SCAD | 0.79 | 0.21 | 0.3 | 0.76 | 0.88 | 0.06 | 0.59 |
| VLCAD | <.0001 | 0.13 | 0.016 | <.0001 | 0.002 | 0.003 | 0.0004 |
| MCAD | 0.27 | 0.01 | 0.39 | 0.19 | 0.42 | 0.66 | 0.88 |
| CPT2 | <.0001 | 0.06 | 0.07 | 0.07 | 0.04 | 0.122 | 0.04 |
| CRAT | <.0001 | 0.056 | 0.05 | 0.06 | 0.05 | 0.002 | 0.23 |
| ACOT3/4 | <.0001 | 0.33 | 0.01 | 0.05 | 0.13 | 0.27 | 0.12 |
| ACSL3 | <.0001 | 0.002 | 0.13 | 0.01 | 0.07 | 0.9 | 0.08 |

Supplementary Table 6. Statistical analysis for hydroxy-acylcarnitines.

| **ANOVA (Diet)** | | | | | **p Value; Multiple Comparisons (Time)** | | | | | |
| --- | --- | --- | --- | --- | --- | --- | --- | --- | --- | --- |
| **Acylcarnitine** | **Mean Amount (nmol / g tissue)** | | **Standard Deviation** | **P value** | **2** | **6** | **10** | **14** | **18** | **22** |
|  |  |  |  |  |  |  |  |  |  |  |
| C4-OH | AL | 6.49 | 2.64 | <0.001 | 0.01 | 0.06 | 0.00 | 0.16 | 0.16 | 0.16 |
|  | CR | 40.22 | 15.22 |  |  |  |  |  |  |  |
| C6-OH | AL | 0.23 | 0.23 | 0.220 | >0.99 | >0.99 | >0.99 | >0.99 | >0.99 | >0.99 |
|  | CR | 0.06 | 0.04 |  |  |  |  |  |  |  |
| C8:1-OH | AL | 0.25 | 0.14 | 0.005 | 0.47 | >0.99 | 0.42 | 0.73 | >0.99 | >0.99 |
|  | CR | 0.66 | 0.26 |  |  |  |  |  |  |  |
| C10-OH | AL | 13.73 | 4.75 | 0.008 | 0.43 | 0.33 | >0.99 | 0.14 | 0.29 | 0.45 |
|  | CR | 36.43 | 19.84 |  |  |  |  |  |  |  |
| C12-OH | AL | 26.92 | 15.17 | 0.300 | >0.99 | >0.99 | >0.99 | >0.99 | 0.50 | >0.99 |
|  | CR | 18.92 | 11.14 |  |  |  |  |  |  |  |
| C12:1-OH | AL | 2.21 | 0.75 | 0.030 | >0.99 | 0.04 | >0.99 | 0.48 | >0.99 | >0.99 |
|  | CR | 4.99 | 2.80 |  |  |  |  |  |  |  |
| C14-OH | AL | 0.46 | 0.45 | <0.001 | 0.02 | >0.99 | 0.06 | 0.15 | 0.53 | 0.39 |
|  | CR | 2.23 | 1.29 |  |  |  |  |  |  |  |
| C14:1-OH | AL | 0.28 | 0.19 | <0.001 | 0.02 | 0.28 | 0.72 | 0.40 | 0.45 | 0.28 |
|  | CR | 2.54 | 1.53 |  |  |  |  |  |  |  |
| C16-OH | AL | 1.70 | 1.77 | 0.390 | 0.87 | >0.99 | >0.99 | >0.99 | 0.50 | >0.99 |
|  | CR | 2.08 | 1.44 |  |  |  |  |  |  |  |
| C16:1-OH | AL | 0.96 | 0.33 | <0.001 | 0.14 | >0.99 | 0.56 | 0.76 | >0.99 | 0.61 |
|  | CR | 3.00 | 2.63 |  |  |  |  |  |  |  |
| C18-OH | AL | 0.03 | 0.02 | 0.001 | 0.28 | 0.05 | >0.99 | 0.08 | 0.11 | 0.08 |
|  | CR | 0.14 | 0.05 |  |  |  |  |  |  |  |
| C18:1-OH | AL | 0.16 | 0.16 | <0.001 | 0.01 | 0.37 | 0.11 | 0.02 | 0.38 | 0.52 |
|  | CR | 2.18 | 1.61 |  |  |  |  |  |  |  |
| C18:2-OH | AL | 1.08 | 0.87 | 0.390 | <0.001 | 0.04 | >0.99 | >0.99 | >0.99 | >0.99 |
|  | CR | 1.24 | 0.69 |  |  |  |  |  |  |  |
| C20-OH | AL | 0.20 | 0.15 | 0.170 | >0.99 | >0.99 | >0.99 | >0.99 | 0.46 | >0.99 |
|  | CR | 0.35 | 0.28 |  |  |  |  |  |  |  |

Supplementary Table 7. Statistical analysis for acyl-CoA.

| **ANOVA (Diet)** | |  |  |  | **p Value; Multiple Comparisons (Time)** | | | | | |
| --- | --- | --- | --- | --- | --- | --- | --- | --- | --- | --- |
| **Acylcarnitine** | **Mean Amount (nmol / g tissue)** | | **Standard Deviation** | **P value** | **2** | **6** | **10** | **14** | **18** | **22** |
|  |  |  |  |  |  |  |  |  |  |  |
| Free-CoA (C0)** | AL | 27.21 | 26.68 | 0.020 | 0.080 | 0.005 | 0.005 | <0.001 | 0.009 | 0.020 |
|  | CR | 172.60 | 136.90 |  |  |  |  |  |  |  |
| Acetyl-CoA (C2) | AL | 1.41 | 0.92 | <0.001 | 0.002 | 0.170 | 0.740 | 0.050 | 0.006 | 0.001 |
|  | CR | 29.51 | 8.49 |  |  |  |  |  |  |  |
| Butyryl-CoA (C4) | AL | 0.27 | 0.22 | <0.001 | 0.120 | 0.250 | >0.99 | 0.030 | 0.090 | 0.160 |
|  | CR | 7.19 | 2.88 |  |  |  |  |  |  |  |
| Hexanoyl-CoA (C6) | AL | 0.38 | 0.25 | 0.040 | 0.860 | 0.130 | >0.99 | 0.140 | 0.380 | 0.500 |
|  | CR | 2.64 | 1.29 |  |  |  |  |  |  |  |
| Palmitoyl-CoA (C16) | AL | 14.34 | 26.36 | 0.540 | >0.99 | >0.99 | >0.99 | >0.99 | 0.690 | >0.99 |
|  | CR | 15.04 | 17.44 |  |  |  |  |  |  |  |
| Malonyl-CoA | AL | 0.12 | 0.07 | 0.430 | >0.99 | >0.99 | >0.99 | >0.99 | 0.120 | >0.99 |
|  | CR | 0.09 | 0.05 |  |  |  |  |  |  |  |
| Octanoyl-CoA (C8) | AL | 1.22 | 0.69 | 0.030 | >0.99 | 0.300 | >0.99 | >0.99 | 0.030 | 0.760 |
|  | CR | 3.34 | 1.89 |  |  |  |  |  |  |  |
| 3-hydroxybutyryl-CoA | AL | 1.21 | 0.65 | 0.008 | 0.740 | 0.260 | 0.160 | 0.030 | 0.002 | 0.400 |
|  | CR | 5.41 | 4.27 |  |  |  |  |  |  |  |

Supplementary Table 8. Statistical analysis light versus dark for acylcarnitines.

|  | AL light versus dark | CR light versus dark | light AL versus CR | dark AL versus CR |
| --- | --- | --- | --- | --- |
| C0 | 0.332016196 | 0.139717965 | 0.000809226 | 5.00482E-05 |
| C2 | 0.332016196 | 0.139717965 | 0.000809226 | 5.00482E-05 |
| C4 | 0.332016196 | 0.139717965 | 0.000809226 | 5.00482E-05 |
| C4-OH | 0.316858872 | 0.531306958 | 1.01157E-09 | 0.000199736 |
| C6 | 0.272956861 | 0.063498845 | 0.039953082 | 0.036483694 |
| C6-OH | 0.080825541 | 0.123713259 | 0.152132141 | 0.147254359 |
| C8:1 | 0.929843978 | 0.335172728 | 0.013932859 | 0.000586933 |
| C8:1-OH | 0.775218867 | 0.032206628 | 0.000919251 | 0.148421249 |
| C10 | 0.951832099 | 0.079198108 | 0.711245545 | 0.126714048 |
| C10-OH | 0.746086742 | 0.686900985 | 0.002896739 | 0.060867036 |
| C12 | 0.448840511 | 0.580979102 | 0.054772925 | 0.247192277 |
| C12-OH | 0.812676586 | 0.066411953 | 0.729407371 | 0.061034558 |
| C12:1 | 0.099175545 | 0.113683633 | 0.487205431 | 0.856428473 |
| C12:1-OH | 0.923865567 | 0.075933808 | 0.016898417 | 0.61666605 |
| C14 | 0.191400534 | 0.013211262 | 0.016391934 | 0.017557903 |
| C14-OH | 0.551705103 | 0.906575369 | 0.00649311 | 4.39332E-05 |
| C14:1 | 0.31132967 | 0.376347985 | 0.000768151 | 0.000916062 |
| C14:1-OH | 0.016367841 | 0.328801574 | 0.000486123 | 4.00839E-05 |
| C16 | 0.959124634 | 0.430741917 | 0.593123726 | 0.806943359 |
| C16-OH | 0.007382414 | 0.308579728 | 0.085150827 | 0.669257639 |
| C16:1 | 0.207497999 | 0.980159297 | 0.326246893 | 0.984080005 |
| C16:1-OH | 0.650544105 | 0.210813022 | 0.019032348 | 0.005279693 |
| C18 | 0.765851659 | 0.192361815 | 0.07101032 | 0.192866211 |
| C18-OH | 0.390401024 | 0.021486236 | 0.001091076 | 0.000213027 |
| C18:1 | 0.85214033 | 0.3126476 | 0.607136009 | 0.653586092 |
| C18:1-OH | 0.196597493 | 0.296092921 | 0.001196347 | 1.42397E-06 |
| C18:2 | 0.614049583 | 0.866827749 | 0.004769835 | 0.003977527 |
| C18:2-OH | 0.021767144 | 0.426565899 | 0.101355311 | 0.780909277 |

KEY RESOURCES TABLE

| REAGENT OR RESOURCE | SOURCE | IDENTIFIER |
| --- | --- | --- |
| Antibodies |  |  |
| ACAA2 | Santa Cruz | Cat# sc-100847 |
| Acetylated-Lysine | Cell Signaling Technology | Cat# 944S |
| ACSS2 | Santa Cruz | Cat# sc-398559 |
| ACOT4 | Invitrogen | Cat# PA5-51453 |
| ACOX1 | Santa Cruz | Cat# sc-517306 |
| ACSL3 | Santa Cruz | Cat# sc-166374 |
| β-actin Monoclonal Antibody | Sigma | Cat# A5441 |
| Catalase | Santa Cruz | Cat# sc-34285 |
| CPT2 | Santa Cruz | Cat# sc-377294 |
| CPT1 | Santa Cruz | Cat# sc-393070 |
| CRAT | Abcam | Cat# ab153750 |
| GAPDH | Cell Signaling Technology | Cat# 5174S |
| HADHB | Santa Cruz | Cat# sc-271495 |
| HADHSC | Santa Cruz | Cat# sc-376525 |
| HystoneH3 | Abcam | Cat# ab8580 |
| LCAD | Invitrogen | Cat# PA5-82450 |
| MCAD | Santa Cruz | Cat# sc-271931 |
| SCAD | Santa Cruz | Cat# sc-365953 |
| Phospho-S6 Ribosomal Protein (Ser235/236) | Cell Signaling Technology | Cat# 4858S |
| Ribosomal Protein S6 Antibody (C-8) | Santa Cruz | Cat# sc-74459 |
| VDAC1 | Santa Cruz | Cat# sc-390996 |
| VLCAD | Santa Cruz | Cat# sc-376239 |
| Anti-rabbit IgG, HRP-linked Antibody | Cell Signaling Technology | Cat# 7074; RRID: AB_2099233 |
| Anti-mouse IgG, HRP-linked Antibody | Cell Signaling Technology | Cat# 7076; RRID: AB_330924 |
| Anti-goat IgG, HRP-linked Antibody | Santa Cruz | Cat# sc-2354 |
| Experimental Models: Organisms/Strains |  |  |
| C57BL/6J mice | Jackson Laboratory | 000664 |
| Oligonucleotides |  |  |
| Bmal1 primers | IDT | Fwd 5’ CACTGTCCCAGGCATTCCA 3’  Rev 5’ TTCCTCCGCGATCATTCG 3’ |
| Per1 primers | IDT | Fwd 5’ AGGTGGCTTTCGTGTTGG 3’  Rev 5’ CAATCGATGGATCTGCTCTGAG 3’ |
| Per2 primers | IDT | Fwd 5’ AGGCACCTCCAACATGCAA 3’  Rev 5’ GGATGCCCCGCTTCTAGAC 3’ |
| AcadL primers | IDT | Fwd 5’ AGGGAAGAGCAAGCGTACTCCC 3’  Rev 5’ TCTGTCATGGCTATGGCACC 3’ |
| AcadVL primers | IDT | Fwd 5’ ATTCCGGATCTTTGAAGGGGC 3’  Rev 5’ CTGGGTGGACAATCCCTGAC 3’ |
| Acot4 primers | IDT | Fwd 5’ ACATCCTGGAACTTGCCATGTA 3’  Rev 5’ GGCCGAGCCTTTAATCCTATC 3’ |
| Acot3 primers | IDT | Fwd 5’ CACCGCTACCTGGAATGTAAT 3’  Rev 5’ CCTTCCAAGCCTCTTTCTAGTC 3’ |
| Cpt1a primers | IDT | Fwd 5’ GGCCATCTGTGGGAGTATGT 3’  Rev 5’ CTCCTGGAATGTCCCACTGT 3’ |
| Cpt2 primers | IDT | Fwd 5’ ACAGCCAGTTCAGGAAGACA 3’  Rev 5’ TGAGATGTAGCTGGTGTGCT 3’ |
| Crat primers | IDT | Fwd 5’ CAGTGAGGGCTCTCAACCTC 3’  Rev 5’ CAAGCTCCCTGAACCAGAGAC 3’ |
| Hadhb primers | IDT | Fwd 5’ TTCCCCACAGGCAGATTTCAG 3’  Rev 5’ GCAGAAATGGAATGCGGACC 3’ |
| AcadS primers | IDT | Fwd 5’ GCTTGGGCAACTTTGCAGTA 3’  Rev 5’ CCAGCGCCTTATGTCTGCTA 3’ |
| AcadM primers | IDT | Fwd 5’ GATGACGGGTCCAAGCAGAA 3’  Rev 5’ CCGAGACCCTCCCTCATCTA 3’ |
| Slc25a20 primers | IDT | Fwd 5’ CCCCATGAGAGCACTCAGTTT 3’  Rev 5’ CCGCTGAAGTGTCCCTGAAT 3’ |
| Hmgcs2 primers | IDT | Fwd 5’ CGCAGAAGTAGAATCCTCTC 3’  Rev 5’ GAAGACTAGAACCCCTTGTC 3’ |
| 18s rRNA primers | IDT | Fwd 5’ GCTTAATTTGACTCAACACGGGA 3’  Rev 5’ AGCTATCAATCTGTCAATCCTGTC 3’ |
| Chemicals, Peptides and Recombinant Proteins |  |  |
| TRIzol™ Reagent | Invitrogen™ | Cat# 15596018 |
| Chloroform | Fisher | Cat# C298-500 |
| Laboratory grade 2-Isopropanol | Fisher | Cat# A415-4 |
| Ethyl alcohol, absolute, 200 proof | Acros | Cat# 61509-0020 |
| UltraPure DEPC Treated Water | Invitrogen™ | Cat# 750023 |
| Random Hexamers (50 µM) | Invitrogen™ | Cat# N8080127 |
| SuperScript™ IV Reverse Transcriptase | Invitrogen™ | Cat# 18090200 |
| UltraPure™ DEPC-Treated Water | Invitrogen™ | Cat# 750023 |
| dNTP 100mM | BioBasic | Cat# DD058 |
| iTaq™ Universal SYBR® Green Supermix | Bio-Rad | Cat# 1725125 |
| Methanol | Fisher | Cat# A412-4 |
| Tris Base | Fisher | Cat# BP152-5 |
| Glycine | Fisher | Cat# BP381-5 |
| EDTA | BioBasic | Cat# EB0436 |
| SDS | BioBasic | Cat# SB0485 |
| MOPS Free Acid | BioBasic | Cat# MB0360 |
| Ponceau S Solution | Sigma | Cat# P7170-1L |
| Bovine Serum Albumin | BioBasic | Cat# AD0023 |
| Non-Fat Dry Milk powder | Value Time | N/A |
| Clarity Western ECL Substrate | Bio-Rad | Cat# 1705061 |
| PVDF Transfer membrane | Thermo Scientific | Cat# 88518 |
| Bovine Gamma globulin, Standard, 2mg/ml | Thermo Scientific | Cat# 23212 |
| Sodium Chloride | Fisher | Cat# S671-3 |
| Precision Plus Protein™ Kaleidoscope™ Prestained Protein Standards | Bio-Rad | Cat# 1610395 |
| Bio-Rad Protein Assay Dye | Bio-Rad | Cat# 5000006 |
| Software and Algorithms |  |  |
| GraphPad Prism 7 | GraphPad | RRID:SCR_002798 |
| Image Studio Lite for western blots | LI-COR Biosciences | N/A |
| Other |  |  |
| Formulab Diet, Irradiated | LabDiet | Cat# 5008 |
| Precision Xtra Blood Glucose and Ketone Meter | Abbott | Item# 98814-65 |
| Precision Xtra Blood Ketone Test Strips | Abbott | Lot# 75001 |
| CFX Connect™ Real-Time PCR Detection System | Bio-Rad | Cat# 1855200 |
| Odyssey® Fc Imaging System | LI-COR Biosciences | N/A |
| XCell SureLock Mini-Cell Electrophoresis System | Invitrogen™ | Cat# EI0001 |
| PowerPac Universal Power Supply | Bio-Rad | Cat# 1645070 |
| Nanodrop 2000 | Thermofisher Scientific | N/A |

CONTACT FOR REAGENT AND RESOURCE SHARING

Requests for further information or reagents should be directed to the Lead Contact, Roman V. Kondratov ([r.kondratov@csuohio.edu](mailto:r.kondratov@csuohio.edu)).

EXPERIMENTAL MODEL AND SUBJECT DETAILS

Animals

All experiments involving animals were conducted in accordance with Federal and University guidelines, all procedures were approved by IACUC, Cleveland State University. C57BL/6J mice were bred in-house at Cleveland State University. Mice were maintained on 12 h light: 12 h dark cycle (LD12:12) with lights on at 7am and lights off at 7pm. Mice used in the experiments were 12-16 weeks of age at the start of the experiments. Animals were maintained in groups of three-four animals per cage (Micro-VENT System Caging, # PC7115HT; Overall dimension: 7 3/4"W x 12"D x 6 1/2"H, Allentown, NJ) throughout the experiment. Temperature for animal rooms was maintained at 20 ± 5^0^C and humidity between 30-70%. All mice were fed 5008 LabDiet (proteins 26.5 %, fat 16.9%, carbohydrates 56.5%). Calorie restriction treatment was implanted gradually with 10% reduction of calorie in the first week, 20% in the second week, and 30% reduction was started in the third week. Calorie restricted animals received food once per day 2 hours after the lights were turned off (ZT14). At 5 months of age liver tissues were collected across 24 hours every 4 hours and immediately frozen and kept at -80°C for subsequent processing.

METHOD DETAILS

Blood β-hydroxybutyrate

Blood for the analysis was collected through the tail vein nick at time points ZT2, 6, 10, 14, 18 and 22. Blood ketones were measured as β-hydroxybutyrate level using Precision Xtra Blood Glucose and Ketone meter (Abbott Laboratories, IL, USA).

RNA isolation and processing

Total RNA was extracted from frozen liver tissue using TRIzol according to manufacturer’s instructions. Briefly, frozen tissue was mixed with 1ml of TRIzol reagent, homogenized using sonicator, centrifuged at 12,000 rpm for 10 mins. Supernatant from this solution was transferred to new tube and mixed with 200µl chloroform, shaken, and centrifuged at 11,500 rpm for 15 minutes at 4^0^C. The aqueous phase was separated and mixed with equal amount of isopropanol, shaken, and centrifuged at 14,000 rpm for 10 minutes at 4^0^C. The RNA pellet was washed with 1ml of 75% ethanol prior to re-suspending the pellet in 30µl RNase-free water. Quantification of RNA was performed using Nanodrop-2000 and quality of total RNA checked by gel electrophoresis.

Analysis of mRNA expression with Real Time quantitative PCR

After quantification and quality check of total RNA by electrophoresis, 1000ng of total RNA was reverse transcribed using SuperScript IV Reverse Transcriptase. Real Time quantitative PCR was performed using iTaq Universal SYBR Green Supermix (Bio-Rad, Hercules, CA) on CFX Connect Real Time PCR detection instrument. For list and details of primers, refer to STAR METHODS. 18S rRNA expression levels were used for normalization. Fold change was determined by ΔΔCt method.

Analysis of protein expression with Western Blotting

Total liver lysates were prepared with Cell Signaling Buffer (1M Tris Base pH 7.5, 5M NaCl, 0.5M EGTA, 0.5M EDTA, Triton-X, 0.1M Na_4_P_2_O_7_, 1M β-glycerophosphate, 1M Na_3_VO_4_) containing protease and phosphatase inhibitor cocktails (Sigma). For fractionated protein extracts 30mg of liver was homogenized with Dounce homogenizer in Buffer A (10mM HEPES, 1.5mM MgCl2, 10mM KCl, 0.5mM DTT, 0.05% NP40, 0.1M Na4P2O7, 1M β-glycerophosphate, 1M Na3VO4 and protease and phosphatase inhibitor cocktails) and centrifugated at 3000 rpm 5 min at 40 C. Pellet contained the nuclear, supernatant was centrifugated at 8000 rpm 5 min for separation cytosolic (supernatant) and mitochondria (pellet) fractions. The homogenates complete with SDS loading mix were loaded in 4-12% Bis-Tris NUPAGE gels (Thermo Scientific). After electrophoretic run, proteins were transferred onto PVDF membrane (Thermo Scientific) and blocked in 5% Milk prepared in TBS with 0.1% Tween-20 for 1 hr. Incubation with primary antibodies was done overnight with gentle shaking at 4^0^C. For list of antibodies, please refer to STAR Methods. β-actin was used as internal control. Quantification of images were done using Image Studio Lite software.

Sample Preparation for Acylcarnitine and acyl-CoA Analysis

Internal standard stock solutions were prepared as follows: Acylcarnitine Internal Standard Mix (1520 nM d9-carnitine, 380 nM d3-acetylcarnitine, 76 nM d3-propionylcarnitine, 76 nM d3-butyrylcarnitine, 76 nM d9-isovalerylcarnitine, 76 nM d3-octanoylcarnitine, 76 nM d9-myristoylcarnitine, 152 nM d3-palmitoylcarnitine) in methanol. 13C_2_-acetyl-CoA (1000 ng/mL in 1:1 v/v methanol:5 % acetic acid).

All sample preparation was performed on ice. Mouse liver tissues (~ 40 mg frozen mass stored at -80 °C) were spiked with 10 µL of NSK-B acylcarnitine internal standard mix (Cambridge Isotope Labs) and 10 uL of 1000 ng/mL 13C_2_-acetyl-CoA (Sigma Aldrich). Each tissue was homogenized in 1 mL of “Extraction Matrix” (1:1 v/v) methanol:5 % acetic acid (aq) using a VWR pellet mixer and centrifuged at 12k rpm for 5 min. The supernatant was transferred to a 13 mm glass tube. The remaining tissue was homogenized and centrifuged again in 1 mL of Extraction Matrix. The combined supernatants were dried under N_2_ and reconstituted in 200 µL of Extraction Matrix. 50 µL was aliquoted for acylcarnitine analysis and the remaining kept at -80 °C for acyl-CoA analysis. The 50 µL aliquot was dried under N_2_ and derivatized by addition of 60 µL butanol-1-HCl (Sigma Aldrich) incubated at 80 °C for 20 min. The sample was dried under N_2_ and reconstituted in 60 µL of (80:20:0.1 v/v/v) acetonitrile:water:formic acid. The samples were stored at -80 °C. For analysis, frozen samples were kept on ice and transferred to HPLC vials with 100 µL glass inserts after vortex mixing.

Acylcarnitine Analysis by LC-MS/MS

Analysis was performed using a Shimadzu Nexera UHPLC coupled to a SCIEX QTrap 5500 mass spectrometer. The SCIEX Turbo V ESI source was operated in positive mode with curtain gas set to 40 psi, CAD gas set to high, IS voltage 4500 V, temperature 700 °C, GS1 and GS2 gases at 50 psi and 45 psi, respectively. Compound-specific parameters were optimized by direct infusion of a 1 uM mixture of butylated free carnitine, acetylcarnitine, butyrylcarnitine, octanoylcarnitine, and palmitoylcarnitine in 0.1 % formic acid in acetonitrile. Butylated acylcarnitines fragment similarly producing a common fragment m/z 85 in positive mode. Therefore, the MRM transitions for acylcarnitines of interest were measured by monitoring the parent ion [M+H]+ > m/z 85.^1,2^ Separation was achieved using a Waters XBridge BEH C18 (75 x 2.1 mm, 2.5 um) column heated to 40 °C with flow rate 0.6 mL/min. The autosampler was kept at 4 °C and sample injection volume set to 1 µL. The needle was rinsed externally after each injection with acetonitrile. Gradient elution: 0.1 % formic acid in HPLC-grade water (mobile phase A) and 0.1 % formic acid in acetonitrile (mobile phase B). Time program: 1 min hold at 20 % B, linear increase to 37 % B at 8 min, linear increase to 100 % B at 22 min. Hold at 100 % B for 4 min followed by re-equilibration at starting conditions for 4 min. Total run time was 30 minutes per sample.

Acyl-CoA Analysis by LC-MS/MS

Analysis was performed using a Shimadzu Nexera UHPLC coupled to a SCIEX QTrap 5500 mass spectrometer. The SCIEX Turbo V ESI source was operated in positive mode with curtain gas set to 30 psi, CAD gas set to medium, IS voltage 5000 V, temperature 550 °C, GS1 and GS2 gases at 40 psi. Compound-specific parameters were optimized by direct infusion of a 1000 ng/mL mixture of free-CoA, acetyl-CoA, succinyl-CoA, glutaryl-CoA, acetoacetyl-CoA, propionyl-CoA, octanoyl-CoA, palmitoyl-CoA, octadecenoyl-CoA, and 3-hydroxybutyryl-CoA. Acyl-CoAs produce the common fragment m/z 507. The acyl-CoAs of interest were detected by monitoring the MRM transition of parent ion [M+H]+ to the appropriate fragment ion produced by the loss of m/z 507.^3,4^ Separation was achieved using a Waters Acquity CSH C18 (50 x 2.1 mm, 1.7 um) column heated to 30 °C with flow rate 0.6 mL/min. The autosampler was kept at 4 °C and sample injection volume set to 5 µL. The needle was rinsed externally after each injection with (3:2:1:0.2 v/v/v/v) acetonitrile:isopropanol:water:phosphoric acid. Gradient elution: 100 mM ammonium formate in 98:2 v/v water:acetonitrile (mobile phase A) and 5 mM ammonium formate in 95:5 v/v acetonitrile:water. (mobile phase B). Time program: 1 min hold at 2.5 % B, linear increase to 55 % B at 4 min, linear increase to 98 % B at 6 min. Hold at 98 % B for 2 min followed by re-equilibration at starting conditions for 7 min. Total run time was 15 min per sample.

Quantification and Statistical Analysis

Data for all three diets was analyzed using ordinary two-way ANOVA (multiple comparison correction was done using Bonferroni method). All statistical analysis was performed using GraphPad Prism 7.0 (San Diego, CA). At least three biological replicas have been used for every time point and every diet. Exact number of biological samples for every experiment is reported in Figure legend. Data are shown as Mean ± SD. p ≤ 0.05 was considered as statistically significant difference between the diets.

Detailed methods are provided in the online version of this paper and include the following:

- KEY RESOURCES TABLE
- CONTACT FOR REAGENT AND RESOURCE SHARING
- EXPERIMENTAL MODEL AND SUBJECT DETAILS
- Animals
- METHOD DETAILS
- Blood glucose and β-hydroxybutyrate
- RNA isolation and processing
- Analysis of mRNA expression with Real Time quantitative PCR
- Analysis of protein expression with Western Blotting
- Sample Preparation for Acylcarnitines and Acyl-CoAs Analysis
- Acylcarnitine Analysis by LC-MS/MS

Quantification and Statistical Analysis
